# Impact of using supplemented thylakoids derived from spinach for 12 weeks of high-intensity functional training on adipo-myokines in obese males

**DOI:** 10.3389/fnut.2025.1513681

**Published:** 2025-10-01

**Authors:** Asieh Abbassi-Daloii, Maha Hoteit, Zahra Sadek, Mahboubeh Khak-Rand, Akbar Ramezani, Zhaleh Pashaei, Mahsa Afshar, Kurt A. Escobar, Rashmi Supriya, Ayoub Saeidi, Hassane Zouhal, Ahmad Alkhatib

**Affiliations:** ^1^Department of Exercise Physiology, Am.C., Islamic Azad University, Amol, Iran; ^2^PHENOL Research Group (Public Health Nutrition Program Lebanon), Faculty of Public Health, Lebanese University, Beirut, Lebanon; ^3^Institut National de Santé Publique, d’Epidémiologie Clinique, et de Toxicologie (INSPECT-LB), Beirut, Lebanon; ^4^Department of Primary Care and Population Health, University of Nicosia Medical School, Nicosia, Cyprus; ^5^Faculty of Public Health, Section I, Lebanese University, Beirut, Lebanon; ^6^Laboratory of Motor System, Handicap and Rehabilitation (MOHAR), Faculty of Public Health, Lebanese University, Beirut, Lebanon; ^7^Department of Exercise Physiology, Faculty of Physical Education and Sport Sciences, University of Tabriz, Tabriz, Iran; ^8^Department of Physical Education, Science and Research Branch, Islamic Azad University, Tehran, Iran; ^9^Department of Kinesiology, California State University, Long Beach, CA, United States; ^10^Centre for Health and Exercise Science Research, Hong Kong Baptist University, Kowloon Tong, Hong Kong SAR, China; ^11^Department of Sport, Physical Education and Health, Faculty of Arts and Social Sciences, Hong Kong Baptist University, Kowloon Tong, Hong Kong SAR, China; ^12^Department of Physical Education and Sport Sciences, Faculty of Humanities and Social Sciences, University of Kurdistan, Sanandaj, Iran; ^13^International Institute of Sport Sciences (2I2S), Irodouer, France; ^14^Sport Performance Optimisation Research Laboratory (LR09SEP01), National Center for Medicine and Science (CNMSS), Tunis, Tunisia; ^15^Department of Life and Sport Sciences, School of Health and Life Sciences, Birmingham City University, Birmingham, United Kingdom

**Keywords:** high-intensity functional training, spinach-derived thylakoid, adipokines, insulin resistance, obesity

## Abstract

**Objective:**

This randomized controlled study investigated the independent and combined effects of High-Intensity Functional Training (HIFT) and spinach-derived thylakoid supplementation on adipo-myokines, glycemic control, and lipid profiles in obese males. To compare the effects of HIFT alone, thylakoid supplementation (Thyl) alone, and their combination (HIFT+Thyl) on circulating adipokines (CTRP-2, CTRP-9, GDF-8, GDF-15), insulin resistance, and lipid profiles in obese adult males.

**Methods:**

A total of 68 participants who were obese with BMI: 32.6 ± 2.6 kg/m2 were randomly assigned to four groups (*n* = 17 in each group): thylakoid supplementation (Thyl), HIFT + Placebo High-Intensity Functional Training (HIFT), HIFT + thylakoid supplementation (HIFT+Thyl), and control+Placebo group (C). The training groups (HIFT and HIFT+Thyl) completed a 12-week program of three 60-min sessions per week. Participants in the Thyl and HIFT+Thyl groups dissolved and consumed 5 g/day of spinach extract high in thylakoids (or placebo) for 12 weeks. Baseline and post-intervention measurements included circulating C1Q/TNF or TGF-*β* related proteins (CTRP-2, CTRP-9, GDF-8, GDF-15), insulin resistance (HOMA-IR, plasma glucose, and insulin), lipid profile (HDL-C, LDL-C, triglycerides [TG], total cholesterol [TC]), and body composition (BMI, fat mass [FM], and fat-free mass [FFM]). Randomization was performed using a block randomization method with allocation concealment.

**Results:**

There were significant group × time interactions for all variables (all *p* < 0.001): CTRP-9 (*η*^2^ = 0.6), CTRP-2 (*η*^2^ = 0.7), GDF-8 (*η*^2^ = 0.8), GDF-15 (*η*^2^ = 0.4), BMI (*η*^2^ = 0.45), FM (*η*^2^ = 0.42), HDL-C (*η*^2^ = 0.37), LDL-C (*η*^2^ = 0.34), TC (*η*^2^ = 0.46), TG (*η*^2^ = 0.66), insulin (*η*^2^ = 0.78), glucose (*η*^2^ = 0.5), and HOMA-IR (*η*^2^ = 0.7). Compared with baseline, all interventions (HIFT, Thyl, and HIFT+Thyl) significantly decreased adipokine levels (CTRP-9, CTRP-2, GDF-8, GDF-15), BMI, fat mass, LDL-C, TC, TG, insulin, glucose, and HOMA-IR, while increasing HDL-C (all *p* < 0.05). Post-hoc between-group comparisons showed that HIFT+Thyl resulted in significantly greater improvements in all adipo-myokines, lipid profile, glycemic and insulin control, and body fat compared to Thyl alone (all *p* < 0.05). HIFT and HIFT+Thyl showed comparable reductions in BMI, fat mass, and improvements in lipid profile and insulin sensitivity.

**Conclusion:**

These findings indicate that HIFT combined with spinach-derived thylakoid supplementation significantly decreases circulating adipo-myokines and improves insulin resistance and lipid profiles in obese adults, suggesting a promising lifestyle intervention for obesity management and cardiometabolic disease prevention. Further research is warranted to explore long-term effects and underlying mechanisms.

**Clinical Trial Registration:**

https://irct.behdasht.gov.ir/trial/69048, identifier (IRCT20151228025732N77).

## Introduction

Adipose tissues and skeletal muscles act as active endocrine organs, secreting a variety of proteins, known as adipokines and myokines, which play crucial roles in maintaining cardiometabolic health (1–5). Adipokines, such as adiponectin are involved in regulating fat and glucose metabolism and mediating associated pathological and physiological processes (6). Tumor necrosis factor C1q (C1q/TNF)-related proteins (CTRPs) are members of the adiponectin family, which include CTRP-1 to CTRP-15 (7). While several in-vitro and in-vivo reports linked each CTRP with a specific metabolic pathway of lipid and glucose regulation (8–15), two CTRPs, CTRP-2 and CTRP-9 have been specifically associated with mechanisms of obesity and insulin resistance metabolic dysfunction (16, 17). Moreover, the myokines, transforming growth factor-beta (TGF-*β*) members, such as Growth differentiation factor 8 & 15 (GDF-8 & 15), are associated with pathological conditions such as obesity and diabetes. It has been demonstrated that baseline levels of myostatin (GDF-8) are elevated in skeletal muscle in individuals with obesity (18). *In vitro* and *in vivo* evidence suggests that GDF-8 affects protein synthesis by acting on the Akt/mTOR pathway and acting as a negative regulator (19, 20). Knockout/genetic deletion of GDF-8 in mice is accompanied by increased skeletal muscle mass and improvement in insulin resistance and lipid profile such as increased HDL-C and decreased LDL-C (21). GDF-15 is expressed in and secreted by various cell types including both adipocytes and skeletal muscles in response to cellular stress (22). In addition to its new function as a component that controls energy metabolism and homeostasis, GDF-15 is thought to have protective effects on a number of organs (19, 20). Mice overexpressing GDF-15 have been shown to have altered appetite and body weight management (21, 22). However, elevated GDF-15 levels have also been implicated in a number of pathophysiological conditions related to muscle atrophy and cachexia in intensive care patients (23), non-fatty liver disease in overweight adolescents (24), and lower physical performance in older adults (25). The paradoxical role of GDF-15 in obesity treatment is still a matter of research interest (26).

Regular physical activity is well established as an effective prevention and management of obesity and cardio metabolic diseases (27). High-Intensity Functional Training, is a modern fitness method that focuses on diverse, practical exercises performed at high intensity such as single-mode aerobic activities (e.g., running, cycling, rowing), body weight movements (e.g., squats, push-ups), and strength-based exercises (e.g., shoulder press, snatch, deadlift) (28). HIFT trains both aerobic and anaerobic systems and elicits greater muscle recruitment than repetitive aerobic exercises, and a degree of adaptability various fitness levels and potential adherence (28). Limited recent interventions with HIFT in adults with obesity, diabetes and metabolic syndrome, lasting between 6 and 12 weeks, have reported promising improvements in body composition and lipid profile, insulin resistance and physical fitness (29–31). However, there are no reports about levels of adiponectin and TGF-*β* members, and associated mechanisms in individuals with obesity in response to HIFT. Whereas, moderate exercise showed conflicting results on CTRP-2 and CTRP-9 levels in patients with obesity complications (32, 33). Twelve weeks of resistance training have either decreased plasma myostatin GD-8 levels in type-2 diabetes (T2D) patients or increased myofibril biopsied levels in healthy men (34). Furthermore, an increase in GDF-15 level was accompanied by an improvement in metabolic factors following aerobic and resistance training (35–38). Therefore, it would be important to know whether and how HIFT type intervention encompassing different types of aerobic, resistance and functional, would affect the adipokines like CTRP-2 & 9 and GDF-8 & 15.

Previously reported effectiveness of a combined exercise intervention with functional foods, especially green leafy vegetables has been well documented in a variety of high-risk populations (39). In the context of ameliorating obesity and associated risk of diabetes, there has been some recent evidence of spinach derived thylakoids benefits on appetite suppression, improved glucose, insulin and lipid profiles in overweight individuals (40–42). However, there remains a question whether and how thylakoid- rich spinach consumption affects Adipo-Myokine.

Despite growing evidence on the individual benefits of HIFT and thylakoids, a notable research gap exists regarding their combined effects on adipo-myokines and comprehensive metabolic health markers in obese populations. Specifically, the interplay between these interventions and their impact on specific adipokines (CTRP-2 & 9) and myokines (GDF-8 & 15) remains underexplored. Therefore, this study aimed to investigate the independent and combined effects of HIFT, spinach-derived thylakoid supplementation (Thyl), or their combination (HIFT+Thyl) on obesity-related adipo- and myokines (CTRP-2 & 9 and GDF-8 & 15), as well as their impact on glycemic control, lipid profile, and cardiorespiratory fitness in men with obesity. We hypothesized that the combined HIFT+Thyl intervention would result in greater improvements in these primary outcomes compared to either intervention alone or control.

## Methods

### Experimental design and participants

This randomized controlled trial adopted a parallel design and involved 100 male adults with obesity who volunteered to participate. Ultimatesubjly, 68 individuals meeting the inclusion criteria were included in the study. The participants had a mean age of 27.6 ± 8.4 years, mean height of 168.4 ± 2.6 cm, mean body mass of 95.7 ± 3.8 kg, and a mean Body Mass Index (BMI) of 32.6 ± 1.6 kg/m^2^.

Inclusion criteria required participants to have obesity, defined by a BMI greater than 30 kg/m^2^, no engagement in regular physical activity, and no alcohol consumption within the 6 months preceding the study. The exclusion criteria were physical limitations, joint problems, endocrine, metabolic, and cardiovascular illnesses, as well as the use of prescription drugs and supplements that may interfere with the metabolic processes of muscle and adipose tissue. Individuals taking over-the-counter medications containing caffeine, protein, etc., were also excluded.

All participants had a familiarization session 1 week prior to the start of the intervention programs, when study protocols were clearly discussed. They completed written informed consent and a Physical Activity Readiness Questionnaire (PAR-Q) (43). The PAR-Q is a widely used screening tool for physical activity readiness, and its validity and reliability have been well-established in various populations. The participants’ diet was monitored using a 24-h food recall questionnaire. The Research and Ethics Committee of the Islamic Azad University (Damghan Branch) approved all procedures of this study (Ethics code: IR.IAU.DAMGHAN.REC.1401.034). The research was also registered at the Iranian Registry of Clinical Trials^1^ with code IRCTID: IRCT20151228025732N77. All procedures were performed according to the latest revision of the Declaration of Helsinki (44).

**Figure 1:**
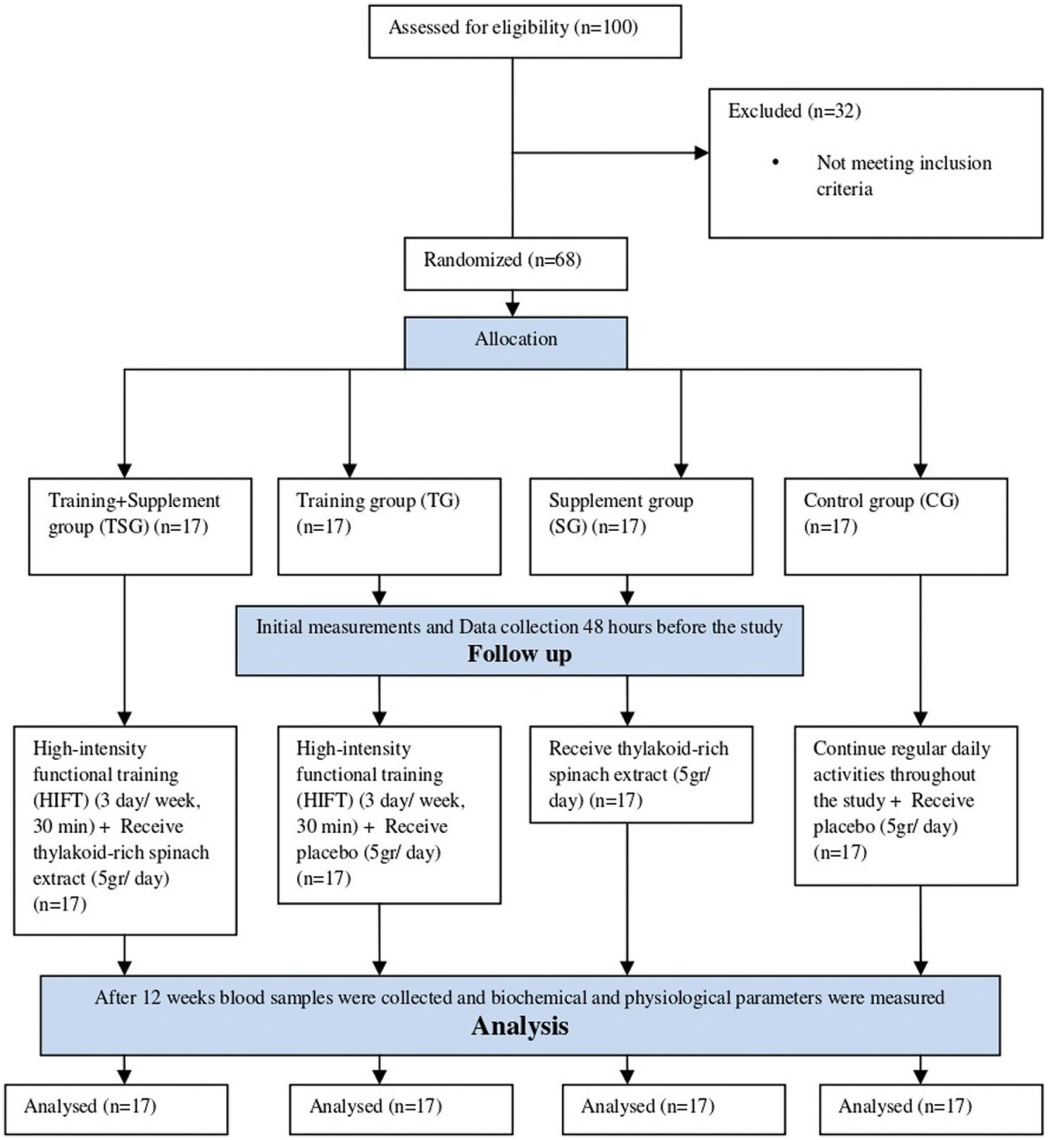
CONSORT flow diagram.

Participants underwent a physical assessment conducted by an examinator during the initial visit. Subsequently, baseline assessments, including anthropometry, body composition, cardiorespiratory, and blood tests, were conducted during a second visit. These evaluations occurred at two stages: baseline and after the 12-week training period. Post-tests was place in all groups 48 h following the final session, and baseline evaluations were carried out 48 h before to the training and/or supplementation regimens. Under constant ambient conditions, study measures were routinely taken in the morning and within an hour. Random assignment then placed participants into one of four equal groups: Control (C), Supplement (Thyl), Training (HIFT), and Training + Supplement (HIFT+Thyl) (Figure 1). The randomization procedure involved a computer-generated block randomization list, with a block size of four, ensuring an equal number of participants in each group. Allocation concealment was maintained by using opaque, sealed envelopes prepared by an independent researcher not involved in participant recruitment or data collection. This was a single-blinded study, where participants were unaware of their group assignment (placebo or thylakoid), but researchers and trainers were not blinded to the training intervention. A similar diet was followed by participants in the intervention protocols 48 h prior to the baseline and final measures.

However, 8 participants withdrew during the study due to reasons such as poor physical fitness, joint weakness, and others, resulting in 15 participants in each group. The sample size was calculated *a priori* based on detecting a clinically meaningful difference in adiponectin levels. Assuming an effect size (Cohen’s d) of 0.8 for adiponectin, with an alpha level of 0.05 and a power of 90%, it was determined that 14 participants per group would be required. Allowing for a potential dropout rate of 20%, we aimed to recruit 17 participants per group, totaling 68 participants. After baseline measurements, the HIFT group commenced the 12-week training program (3 sessions per week), the Thyl group initiated the 12-week supplementation intervention, and the HFT + Thyl group commenced both training and supplementation. The control group maintained their existing lifestyles throughout the 12-week of research.

### Baseline assessments

#### Assessment of body composition

BMI (kg/m^2^) was calculated by measuring body mass (kg) and height (m). To determine fat-free mass (FFM) and fat mass (FM), a bio-electrical impedance analyzer from Medigate Company Inc. (Dan-dong Gunpo, Korea) was utilized.

Before every test, the gas analyzer system (Metalyzer 3B analyzer, Cortex: biophysics, GMbH, Germany) was calibrated in accordance with manufacturer specifications. An electronic sphygmomanometer (Kenz BPM AM 300P CE, Japan) was used to measure blood pressure, and a Polar V800 heart monitor (Finland) was used to record heart rate.

#### Preparation of spinach thylakoids and placebo

Following established techniques (45–47), the thylakoid supplement was prepared from a fresh baby spinach leaf in the laboratory of Islamic Azad University, following the procedure outlined by Emerk et al. (47).

The process involved washing and soaking fresh spinach leaves in cold water after removing stems and veins. The homogenized spinach leaves were then filtered, and the filtrate was diluted and adjusted to pH 4.7 using hydrochloric acid (HCl). After standing in the cold for 4 h, the thylakoids precipitated, forming a green precipitate. The filtrate thylakoids were collected through centrifugation, washed, freeze and dried to produce a green chloroplast powder adjusted to pH 7.0.

The placebo, resembling the thylakoid powder, was made of edible green maize starch with kiwi flavor extract. Corn starch was chosen for its adaptability and ease of modification.

The specified amount, 5 grams, of the material was evenly distributed and enclosed in identical sachets and participants dissolved and consumed the contents of 1 sachet in water 30 min before lunch. The timing of 30 min before lunch was chosen based on previous research demonstrating optimal thylakoid-induced satiety and appetite suppression when consumed prior to a meal, allowing for sufficient gastrointestinal transit and release of satiety hormones.

To ensure compliance, participants were reminded through weekly phone calls, daily text messages, and a supplement consumption chart turned in at each visit. Compliance was assessed by counting leftover sachets, with participants considered adherent if they consumed 80% or more of the extracts. For the training groups, adherence was monitored by attendance records at each session, and participants were considered compliant if they completed at least 80% of the prescribed training sessions.

#### High-intensity functional training (HIFT)

A certified trainer implemented the High-Intensity Functional Training (HIFT) protocol, comprising 36 sessions over 3 days per week, each lasting up to 60 min. Participants were familiarized with the training movements in the initial two sessions. From the third session onward, HIFT sessions involved 10–15 min of stretching and warm-up, followed by 10–20 min of technique practice. The core of each session consisted of a high-intensity workout lasting 5–30 min, known as the Workout of the Day (WOD). Modalities included aerobic activities (e.g., running, jumping rope), body weight movements (e.g., squats, pull-ups, pushups), and resistance-based exercises (e.g., front squats, kettlebell swings). The CrossFit training template was employed, ensuring constant variation in exercise programming. Training loads were adapted to each participant’s physical conditions. The average duration for each workout session and the overall mean workout time per week were computed for the HIFT groups.

Participants aimed to complete each workout as quickly as possible or achieve as many repetitions as possible. Metrics such as time to completion, repetitions, and any modifications made were recorded for each participant, along with the weight used.

Detailed Training Protocol: The HIFT program followed a structured progression, adapting loads and complexity based on individual participant’s fitness levels, ensuring adherence to the principles of specificity (exercises mimicking functional movements and targeting energy systems relevant to HIFT), individualization (adjusting intensity and volume to individual capacities), and progressive overload (gradually increasing demands over the 12 weeks). The full program for all 36 sessions, including specific WODs, exercise variations, prescribed repetitions/times, and load progression guidelines, will be provided in a supplementary file.

#### Blood biomarkers

Blood samples were drawn from the right antecubital vein after a fast 12 h and 72 h before the first workout and 72 h after the last workout. The samples were collected in EDTA-containing tubes, centrifuged for 10 min at 3000 rpm, and then kept at −70°C until additional testing was done. The testing methods were always conducted in the same manner and under the same time constraints, which were 8 to 10 am. Plasma glucose levels were measured using a colorimetric enzymatic kit (Parsazmun, Tehran, Iran) with a sensitivity of 5 mg/dL.

- Insulin resistance was evaluated using the homeostasis model of assessment for insulin resistance (HOMA) calculated as follows: HOMA-IR = 22.5 μmol/(fasting plasma insulin × fasting plasma glucose).
- Plasma total cholesterol (TC) and triglyceride (TG) levels were measured by enzymatic methods (CHOD-PAP).
- High-density cholesterol (HDL-C) and low-density cholesterol (LDL-C) were determined using a photometric method (Pars Testee’s Quantitative Detection kit, Tehran, Iran) with a coefficient and sensitivity of 1.8% and 1 mg/dL, and 1.2% and 1 mg/dL, respectively.
- Insulin levels were measured with an ELISA kit (Demeditec, Germany) with a sensitivity of 1 ng/mL and within-coefficients of variation between 5.1 and 8.4%.
- Plasma CTRP-9 was quantified using an enzyme-linked immunosorbent assay (ELISA) kit (Aviscera Bioscience, USA; Catalogue No: SK00081-02, Sensitivity: 1 ng/mL, Intra-CV = 4%, inter-CV = 8%).
- CTRP-2 was measured with an ELISA kit (MyBioSource, San Diego, CA, USA) with a minimum detectable dose (MDD) of 0.039 ng/mL and detection range of 0.156–10 ng/mL (intra-assay CV: < 8%, inter-assay < 10%).
- GDF-15 levels were determined using an ELISA kit (Thermo Scientific, Frederick, MD, USA) with a sensitivity of 2 pg/mL and a detection range of 1.10–800 pg/mL (intra-assay CV < 10%, inter-assay CV < 12%).
- Plasma GDF-8 was measured with an ELISA kit (R&D Systems, USA; Catalogue No: DGDF80, Sensitivity: 5.32 pg/mL, Intra-CV = 5.4%, inter-CV = 6%).

### Statistical analysis

The study employed descriptive statistics (means ± standard deviation) to summarize the data, with the normality of the data assessed using the Shapiro–Wilk test. Baseline data across all groups were evaluated using one-way ANOVA and Fisher LSD post-hoc tests. To assess interactions between groups (C, Thyl, HIFT, HIFT+Thyl) and time (pre and post), a two-way ANOVA repeated measures test was conducted. Tukey’s *post hoc* test and pairwise comparisons were employed when ANOVA detected significant differences. Effect sizes (ES) were reported in terms of partial eta-squared, categorized as trivial (< 0.2), small (0.2–0.6), moderate (0.6–1.2), large (1.2–2.0), and very large (2.0–4.0). Statistical analysis was carried out using SPSS software (version 24), with the significance level set at (*p* < 0.05).

## Results

### Plasma adipokines levels

Plasma adipokines variables showed significant group x time interactions for CTRP-9 (*η*^2^ = 0.6, *p* < 0.001), CTRP-2 (*η*^2^ = 0.7, *p* < 0.001), GDF-8 (*η*^2^ = 0.8, *p* < 0.001) and GDF-15 (*η*^2^ = 0.4, *p* < 0.001) (Table 1). Within-group analysis revealed that levels of CTRP-2, CTRP-9, GDF-8, and GDF-15 significantly decreased in the HIFT and HIFT+Thyl groups (all *p* < 0.05), but not in the control group (all *p* > 0.05). The Thyl group also showed significant decreases in CTRP-2, CTRP-9, GDF-8, and GDF-15 (all *p* < 0.05).

**Table 1:** Mean, standard deviations (±) and effect size (*η*^2^) of adipokines.

Adipokines	Group	Pre-training Mean (±SD)	Post-training Mean (±SD)	*p* values (*η*^2^)		
				Time	G × T interaction	Group
CTRP-9 (ng/ml)	C	148.9 (±7.9)	153.7 (±10.1)	0.001 (0.6)	0.001 (0.6)$	0.001 (0.6)
	Thyl	145.2 (±9.3)	133.8 (±12.3)*,#			
	HIFT	150.1 (±9.6)	127.1 (±10.9)*,#			
	HIFT+Thyl	153.5 (±9.6)	106.8 (±12.1)*,#			
CTRP-2 (ng/ml)	C	5.1 (±0.5)	4.9 (±0.3)	0.001 (0.8)	0.001 (0.7)$	0.001 (0.8)
	Thyl	4.7 (±0.5)	4 (±0.4)*,#			
	HIFT	5.3 (±0.4)	2.9 (±0.3)*,#			
	HIFT+Thyl	4.8 (±0.4)	2.8 (±0.3)*,#			
GDF-8 (pg/ml)	C	11.8 (±0.8)	12 (±0.5)	0.001 (0.8)	0.001 (0.8)$	0.001 (0.7)
	Thyl	11.9 (±0.7)	10.1 (±0.4)*,#			
	HIFT	12.1 (±0.6)	9.5 (±0.5)*,#			
	HIFT+Thyl	12.0 (±0.9)	8.1 (±0.4)*,#			
GDF-15 (pg/ml)	C	4.0 (±0.4)	3.9 (±0.4)	0.001 (0.6)	0.001 (0.4)$	0.001 (0.4)
	Thyl	3.9 (±0.3)	3.6 (±0.2)*,#			
	HIFT	3.9 (±0.5)	3.1 (±0.5)*,#			
	HIFT+Thyl	4.1 (±0.3)	3.0 (±0.2)*,#			

C1q-TNF related protein 9 (CTRP9), C1q-TNF related protein 2 (CTRP2), growth/differentiation factor 8 (GDF8), growth/differentiation factor 15 (GDF15).

*p < 0.05 compared to Pre-training values within the same group. #*p* < 0.05 compared to the Control group post-intervention. $Significant interaction between time and groups (main ANOVA effects, *p* < 0.05).

Post-hoc multiple comparisons between the intervention groups showed a greater reduction in the HIFT+Thyl group compared to either HIFT or Thyl groups for CTRP-9 (*p* < 0.05) and GDF-8 (*p* < 0.05). Additionally, the HIFT+Thyl group demonstrated a greater reduction in CTRP-2 (*p* < 0.05) and GDF-15 (*p* < 0.05) compared to the Thyl group. No significant difference was found between HIFT+Thyl and HIFT groups for GDF-15 (*p* = 0.4) and CTRP-2 (*p* = 0.5), and no difference between either Thyl or HIFT, or Thyl and Control groups was found for CTRP-9 (*p* = 0.2, *p* = 0.1 respectively).

### Anthropometry, body composition, lipid profile, and cardio-respiratory fitness variables

Obesity related anthropometric, lipid profile and cardiorespiratory variables showed significant group x time interactions for body weight with effect size (0.37), FFM (0.23), FM (0.42), HDL-C (0.37), LDL-C (0.34), TC (0.46) and TG (0.66) (all *p* < 0.05, ANOVA main effects), (Table 2). Within and between group differences were also different (*p* < 0.05, main ANOVA effects for all aforementioned variables). *Post hoc* comparisons showed significant effects of the Thyl, HIFT and HIFT+Thyl interventions, but not in the control group, in all of the measured variables (*p* < 0.05).

**Table 2:** Mean, standard deviations (±) and effect size (*η*^2^) of anthropometry, body composition, lipid profile, insulin resistance and cardio-respiratory fitness variables.

Variables	Group	Pre-training Mean (±SD)	Post-training Mean (±SD)	*p* values (η2)		
				Time	G × T interaction	Group
Weight (Kg)	C	94.33 (±1.82)	93.55 (±2.43)	0.001 (0.63)	0.001 (0.44)$	0.001 (0.37)
	Thyl	93.28 (±2.61)	91.13 (±2.12)*,#			
	HIFT	92.78 (±1.89)	89.19 (±2.37)*,#			
	HIFT+Thyl	94.13 (±1.90)	87.25 (±2.30)*,#			
BMI (kg/m^2^)	C	33.08 (±1.34)	32.87 (±1.44)	0.001 (0.63)	0.001 (0.45)$	0.11 (0.14)
	Thyl	32.66 (±1.37)	31.93 (±0.93)*			
	HIFT	33.22 (±1.07)	31.85 (±1.19)*,#			
	HIFT+Thyl	33.05 (±0.75)	30.68 (±0.95)*,#			
FFM (kg)	C	27.63 (±1.20)	26.54 (±2.25)	0.001 (0.46)	0.001 (0.43)$	0.01 (0.23)
	Thyl	27.09 (±1.81)	29.36 (±0.92)*,#			
	HIFT	26.72 (±1.27)	29.54 (±1.5)*,#			
	HIFT+Thyl	27.18 (±1.77)	30.36 (±1.2)*,#			
Fat percentage (%)	C	30.09 (±1.51)	30.83 (±2.05)	0.001 (0.54)	0.001 (0.46)$	0.001 (0.42)
	Thyl	30.10 (±1.59)	28.08 (±0.79)*,#			
	HIFT	30.36 (±1.50)	26.89 (±0.95)*,#			
	HIFT+Thyl	31.13 (±1.35)	26.62 (±1.21)*,#			
HDL (mg.dl^−1^)	C	39.34 (±1.22)	38.43 (±1.30)	0.001 (0.72)	0.001 (0.73)$	0.001 (0.37)
	Thyl	38.88 (±1.23)	39.74 (±3.99)*			
	HIFT	38.66 (±1.67)	44.51 (±1.34)*,#			
	HIFT+Thyl	38.56 (±1.41)	44.80 (±2.12)*,#			
LDL (mg.dl^−1^)	C	125.22 (±4.47)	125.02 (±4.70)	0.001 (0.91)	0.001 (0.86)$	0.001 (0.34)
	Thyl	125.56 (±5.42)	121.41 (±5.24)*,#			
	HIFT	126.75 (±4.38)	111.20 (±2.92)*,#			
	HIFT+Thyl	127.14 (±3.64)	110.50 (±2.52)*,#			
TC (mg.dl^−1^)	C	226.70 (±5.27)	226.81 (±5.26)	0.001 (0.97)	0.001 (0.96)$	0.001 (0.46)
	Thyl	227.44 (±5.48)	222.09 (±5.19)*,#			
	HIFT	227.81 (±5.29)	207.44 (±4.95)*,#			
	HIFT+Thyl	227.38 (±5.49)	205.23 (±4.77)*,#			
TG (mg.dl^−1^)	C	242.10 (±4.39)	242.81 (±3.85)	0.001 (0.90)	0.001 (0.89)$	0.001 (0.62)
	Thyl	245.83 (±5.93)	242.05 (±5.40)*			
	HIFT	244.58 (±7.48)	217.39 (±9.88)*,#			
	HIFT+Thyl	242.92 (±5.96)	212.85 (±4.64)*,#			
Insulin (ng.ml)^−1^	C	18.81 (±0.67)	19.12 (±0.57)	0.001 (0.84)	0.001 (0.78)$	0.001 (0.77)
	Thyl	18.80 (±0.75)	17.60 (±0.51)*,#			
	HIFT	18.83 (±0.40)	16.12 (±0.43)*,#			
	HIFT+Thyl	19.12 (±0.49)	15.51 (±0.55)*,#			
Glucose (mg.dl^−1^)	C	96.44 (±13.10)	90.73 (±6.43)	0.001 (0.8)	0.001 (0.5)$	0.02 (0.21)
	Thyl	98.99 (±10.71)	84.77 (±4.50)*,#			
	HIFT	99.27 (±5.72)	74.08 (±5.43)*,#			
	HIFT+Thyl	101.63 (±7.13)	71.51 (±7.71)*,#			
HOMA-IR	C	4.48 (±0.70)	4.27 (±0.30)	0.001 (0.87)	0.001 (0.7)$	0.001 (0.44)
	Thyl	4.58 (±0.46)	3.68 (±0.25)*,#			
	HIFT	4.61 (±0.24)	2.94 (±0.26)*,#			
	HIFT+Thyl	4.79 (±0.38)	2.73 (±0.31)*,#			

*p < 0.05 compared to Pre-training values within the same group. #*p* < 0.05 compared to the Control group post-intervention. $Significant interaction between time and groups (main ANOVA effects, *p* < 0.05).

Pairwise comparisons among the control group and all HIFT and HIFT+Thyl groups revealed significant differences in anthropometric, body composition, and cardiorespiratory fitness variables (*p* < 0.05). Notably, the Thyl group displayed no significant differences compared to the control group for BMI (*p* = 0.62), TG (*p* = 0.78), and HDL (*p* = 0.22). However, all pairwise comparisons between Thyl and HIFT groups were statistically significant (*p* < 0.05), except for body weight (*p* = 0.06), BMI (*p* = 0.87), and FFM (*p* = 0.78). No significant differences were identified between HIFT and HIFT+Thyl in body weight (*p* = 0.57), FM (*p* = 0.64), HDL (*p* = 0.78), LDL-C (*p* = 0.68), TC (*p* = 0.31), and TG (*p* = 0.1). Furthermore, HIFT+Thyl showed significant differences compared to Thyl across anthropometry, body composition, lipid profile, and cardiorespiratory fitness variables (*p* < 0.05), except for FFM (*p* = 0.14).

### Insulin, glucose, and HOMA-IR

There was a significant effect for groups, time, and interaction (group × time) for insulin, glucose and HOMA-IR variables (*p* < 0.05), (Table 2). While the intervention significantly affected the aforementioned variables in the Thyl, HIFT, and Thyl+HIFT (*p* < 0.05), there was no significant changes in the control group (*p* > 0.05). The *post hoc* comparisons also showed significant differences post intervention between the control group and all intervention groups (Thyl, HIFT, and Thyl+HIFT) (*p* < 0.01). When comparing HIFT and HIFT+Thyl through pairwise analysis, no significant differences were observed in glucose levels (*p* = 0.33) and HOM-IR (*p* = 0.9). However, the HIFT+Thyl intervention demonstrated significantly lower levels of insulin, glucose, and HOM-IR compared to the Thyl group (all *p* < 0.05).

## Discussion

The primary conclusion of this study reveals that combining High-Intensity Functional Training (HIFT) with spinach-derived thylakoid extract effectively reduces obesity-related adipo-myokines (CTRP-2 & 9 and GDF-8 & 15). Additionally, the intervention improved lipid profiles and glucose homeostasis in men with obesity. These effects, observed at the cellular level, contributed to overall improvements in cardiorespiratory capacity and body composition. The combined approach of HIFT and thylakoid supplementation plays a critical role in mitigating obesity-related risks, particularly Type 2 Diabetes (T2D) and cardiovascular disease.

Recent studies highlight CTRP-9’s role in insulin resistance, where increased concentrations improve glucose homeostasis. A moderate increase in CTRP-9 expression, induced in mice through an adenoviral vector, decreased blood glucose and insulin levels. Conversely, prolonged overexpression of CTRP-9 led to a lean phenotype, while deletion of the CTRP-9 gene resulted in insulin resistance and impaired metabolic function (43). The administration of recombinant CTRP-9 enhanced AMPK activation and fatty acid oxidation, suggesting that upregulation of CTRP-9 may compensate for severe obesity and insulin resistance (44). The current study supports these findings by showing that exercise training and thylakoid supplementation reduce CTRP-9, likely improving body composition and insulin resistance (32).

CTRP-2 levels were also affected by HIFT and thylakoid supplementation. A study by Jerobin et al. indicated that plasma CTRP-2 concentrations increase in response to lipid infusion, suggesting that CTRP-2 plays a role in lipid and energy metabolism (32). Mice lacking CTRP-2 exhibit increased energy expenditure and enhanced lipolytic enzyme expression. In contrast, transgenic mice overexpressing CTRP-2 demonstrate improved free fatty acid (FFA) clearance and higher glucose disposal rates (48). These results suggest that CTRP-2 plays a role in lipid metabolism and glucose homeostasis, and its regulation could be a key factor in managing obesity and related metabolic diseases.

GDF-8, a myokine involved in regulating skeletal muscle mass, has been associated with impaired metabolic health, including elevated BMI, fasting plasma glucose, and dyslipidemia (49, 50). Suppressing GDF-8 in skeletal muscle has shown to improve insulin sensitivity and prevent diabetes development in mouse models (51). Studies have demonstrated that exercise, especially aerobic training, reduces GDF-8 levels, thereby improving insulin sensitivity (45, 46). In this study, GDF-8 levels decreased by around 30% following HIFT and thylakoid supplementation, accompanied by improvements in insulin sensitivity and glucose homeostasis, supporting the notion that GDF-8 may be a crucial mediator of metabolic health in obese individuals.

GDF-15, another key biomarker, is elevated in obesity and associated with inflammation-related diseases. It is suggested that increased glucose and insulin levels in obesity trigger the release of GDF-15, which correlates with body weight, body fat, and triglyceride levels (47). In obese and T2D individuals, increased GDF-15 levels are linked to hyperinsulinemia and adiposity progression (47). Despite these associations, studies show that regular exercise can elevate GDF-15 levels, which correlates with improved insulin sensitivity, fat mass reduction, and enhanced *β*-cell function (37). In this study, we observed a reduction in GDF-15 alongside improvements in metabolic health, including blood lipids, insulin resistance, and body composition. These findings further support the notion that exercise and thylakoid supplementation may exert therapeutic effects by modulating GDF-15 levels, ultimately improving cardiometabolic health.

This study also demonstrated that thylakoid supplementation, particularly in combination with HIFT, had favorable effects on BMI, lipid profile, and markers of cardiometabolic health, including insulin, glucose, HOMA-IR, and peak cardiorespiratory capacity (Table 2). Previous research has shown that thylakoids can improve obesity-related markers, including blood lipids, glucose, and insulin resistance, as well as body composition (41, 42). Over 12 weeks, thylakoid supplementation significantly reduced body weight, waist circumference, LDL-C, fasting blood glucose, and insulin in overweight and obese women (41). In animal studies, thylakoid supplementation decreased food intake, body weight, and body fat in female apo-E mice (52). These effects are thought to be linked to altered hunger perception and energy intake, including a reduction in the desire for palatable foods (41, 53, 54). Thylakoids also inhibit pancreatic lipase, which reduces lipid digestion and absorption, leading to a suppression of appetite and activation of satiety signals (55, 56). Our study aligns with these findings, showing that thylakoid supplementation led to a reduction in body mass, body fat %, and fat mass, along with improvements in metabolic health.

While the results of this study support the beneficial effects of HIFT and thylakoid supplementation on metabolic health, the mechanisms by which these interventions influence plasma adipo-myokines, body composition, and metabolic markers remain unclear. It is essential to investigate whether changes in circulating adipo-myokines are causal, facilitating, or simply reflective of improved body composition following exercise and supplementation in obese individuals. Further studies are needed to explore the specific effects of HIFT and thylakoid supplementation on adipo-myokines and their relationship to metabolic health in obesity.

### Limitations

A significant strength of this study is its randomized controlled design, employing a combined intervention approach to assess synergistic effects. The detailed monitoring of training adherence and supplement consumption further strengthens the internal validity. However, limitations include the relatively small sample size per group, which may limit the generalizability of the findings to a broader obese population. The single-blinded nature of the study for supplementation, while necessary, meant that trainers and researchers were not blinded to the training intervention, which could introduce some bias. Future research should consider larger cohorts, double-blinded designs where feasible, and long-term follow-up to assess the sustainability of these metabolic improvements. Further mechanistic studies are also warranted to fully elucidate the pathways through which HIFT and thylakoids interact to influence adipo-myokines.

## Conclusion

These findings conclusively demonstrate that combining HIFT with spinach-derived thylakoid supplementation is an effective strategy for significantly decreasing circulating adipo-myokines and improving insulin resistance and lipid profiles in adults with obesity. This integrated HIFT-thylakoid approach offers a potent lifestyle intervention, directly supporting its inclusion in comprehensive obesity management and cardiometabolic disease prevention programs. Further randomized controlled trials with larger sample sizes and longer follow-up periods are warranted to confirm these benefits and investigate optimal intervention parameters.

## Data Availability

The original contributions presented in the study are included in the article/supplementary material, further inquiries can be directed to the corresponding author.

## Footnotes

^1^ https://irct.ir/trial/69048
